# Outcomes and Decision-Making Following Out-of-Hospital Cardiac Arrest Within a Multidisciplinary Neuroprognostication Pathway in a Tertiary Cardiac Intensive Care Unit

**DOI:** 10.3390/jcm15135252

**Published:** 2026-07-05

**Authors:** Guilherme Movio, Uzma Sajjad, Dana Prisenznakova, Emma Beadle, Daryl Perilla, Soyun Choi, Lauren Woolford, Marco Mion, Ayush Mohan, Maxwell Damian, Branimir Nevajda, Saneesh Suresh, John R. Davies, Maria Rita Maccaroni, Thomas R. Keeble

**Affiliations:** 1 Essex Cardiothoracic Centre, Mid and South Essex NHS Foundation Trust, Basildon SS16 5NL, UK; gui.movio2@nhs.net (G.M.); uzma.sajjad1@nhs.net (U.S.); dana.prisenznakova@nhs.net (D.P.); emma.beadle4@nhs.net (E.B.); daryl.perilla@nhs.net (D.P.); soyun.choi@nhs.net (S.C.); lauren.woolford@nhs.net (L.W.); m.mion@nhs.net (M.M.); ayush.mohan1@nhs.net (A.M.); maxwell.damian@nhs.net (M.D.); branimir.nevajda@nhs.net (B.N.); saneesh.suresh@nhs.net (S.S.); john.davies32@nhs.net (J.R.D.); maria.maccaroni@nhs.net (M.R.M.); 2 Anglia Ruskin School of Medicine & MTRC, Chelmsford CM1 1SQ, UK

**Keywords:** out-of-hospital cardiac arrest, neuroprognostication, withdrawal of life-sustaining treatment, intensive care, multidisciplinary team, Cerebral Performance Category, post-cardiac arrest care

## Abstract

**Background/Objectives:** Neuroprognostication after out-of-hospital cardiac arrest (OHCA) remains clinically challenging, particularly when withdrawal of life-sustaining treatment (WLST) is considered. International guidelines recommend delayed, multimodal assessment, but real-world descriptions of how this is operationalised within multidisciplinary pathways remain limited. **Methods:** We conducted a single-centre retrospective observational cohort study of adults admitted to a tertiary cardiac arrest centre intensive care unit following OHCA between June 2022 and December 2025. Patients were conveyed according to the British Cardiovascular Intervention Society OHCA pathway; therefore, this was a selected cardiac arrest centre cohort enriched for shockable rhythms and suspected reversible cardiac causes, rather than an unselected OHCA population. Patients who remained unconscious at ≥72 h following a sedation hold entered a structured multidisciplinary team (MDT) neuroprognostication pathway. Outcomes included survival to hospital discharge, Cerebral Performance Category (CPC) at discharge, neuroprognostication investigation use, and timing of WLST. **Results:** Of 406 patients admitted following OHCA, 310 were admitted to ICU and included in the analysis. The cohort was predominantly male (82.3%), with a mean age of 63.8 years; 82.9% had ventricular fibrillation as the initial rhythm. Overall, 182 patients (58.7%) survived to hospital discharge, of whom 160 (87.9%) had a favourable neurological outcome (CPC 1–2). A total of 119 patients entered the neuroprognostication pathway. Of these, 72 underwent WLST after completed MDT review, 10 died before MDT decision-making, and 37 survived to hospital discharge. Among patients undergoing WLST, investigation use was high: CT brain 100%, NSE 91.7%, EEG 90.3%, SSEP 88.9%, and MRI brain 27.8%. Median time to WLST was 5.5 days. **Conclusions:** In this selected tertiary CAC cohort, enriched for shockable rhythms through BCIS pathway-based conveyance, survival to hospital discharge was high and neurological outcomes among survivors were predominantly favourable. Within this setting, delayed, multimodal neuroprognostication and WLST decision-making were operationalised through a structured MDT pathway aligned with contemporary guideline recommendations. These findings provide contemporary real-world benchmark data on pathway implementation for comparable centres seeking to evaluate or develop structured neuroprognostication services.

## 1. Introduction

Out-of-hospital cardiac arrest (OHCA) remains a major cause of mortality and morbidity, with long-term neurological disability worldwide [1,2]. Although advances in pre-hospital resuscitation, coronary reperfusion, and post-cardiac arrest intensive care have improved survival in selected populations, neurological prognostication in comatose survivors remains one of the most challenging aspects of post-cardiac arrest care [3,4]. Early prognostic uncertainty is common, and premature treatment-limitation/withdrawal of life sustaining care decisions risk contributing to a self-fulfilling prophecy [5].

To address this, international guidelines recommend a delayed, structured, and multimodal approach to neuroprognostication in comatose post-cardiac arrest patients [3]. Prognostic assessment should generally be undertaken no earlier than 72 h after return of spontaneous circulation (ROSC), once important confounders such as sedation, metabolic disturbance, and residual effects of temperature management have been excluded [3]. These recommendations emphasise concordance across multiple clinical and investigative findings rather than reliance on any single test [3].

Despite these principles, their implementation in routine clinical practice appears variable, and published real-world data describing how centres operationalise delayed multimodal neuroprognostication, including investigation timing, MDT structure, and how prognostic findings are incorporated into WLST decisions remain limited [6,7]. This is particularly relevant because withdrawal of life-sustaining treatment (WLST) is a major determinant of mortality after OHCA, yet the processes that underpin these decisions are often incompletely described in observational studies [5,8].

Our institution is a tertiary cardiac arrest centre (CAC) with a high-volume cardiac intensive care unit (ICU) that has embedded a structured multidisciplinary team (MDT) neuroprognostication pathway into routine post-cardiac arrest care [9]. This pathway is applied to patients who remain unconscious after a defined period of 72 h and incorporates expertise from intensive care, cardiology, neurology, neuroradiology, neurophysiology, specialist nursing, and allied health professionals to support delayed, multimodal prognostic assessment and decision-making [3].

The primary aim of this study was to describe outcomes for all adult patients admitted to ICU following OHCA at our centre, including number of patients that underwent formal neuroprognostication and neurological outcomes at discharge. The secondary aim was to characterise investigation utilisation, timing, and decision-making within our structured MDT neuroprognostication pathway, with particular focus on patients undergoing WLST for poor neurological prognosis. By reporting ICU-wide outcomes alongside a detailed description of MDT-supported prognostication practice, we aim to provide a contemporary real-world description of pathway implementation and service organisation, rather than to determine the effectiveness of the MDT pathway on patient outcomes.

## 2. Materials and Methods

### 2.1. Study Design and Setting

We conducted a single-centre, retrospective observational cohort study of adult patients admitted to a tertiary ICU following OHCA. The study period extended from 1 June 2022 to 31 December 2025, corresponding to the introduction and routine operation of a structured MDT neuroprognostication pathway within the unit.

The study was performed at a regional CAC providing 24-h access to interventional cardiology, advanced neuroimaging, neurophysiology, and specialist intensive care [10]. The centre receives patients via established British Cardiovascular Intervention Society (BCIS) pre-hospital conveyance algorithm that prioritises patients with an initial shockable arrest rhythms or electrocardiographic evidence of acute coronary occlusion (STE on ECG) following return of spontaneous circulation (ROSC). As a result, the study cohort reflects a selected population enriched by initial shockable rhythms and STEMI, rather than an unselected OHCA population [11,12]. This selection pathway is important when interpreting survival and neurological outcomes, as patients conveyed to a tertiary cardiac arrest centre through this route are not representative of the wider OHCA population.

### 2.2. Patient Population

Adult patients (aged ≥ 17 years) admitted to the ICU following sustained ROSC after OHCA (presumed cardiac or medical aetiology) were eligible for inclusion. All included patients received standard post-cardiac arrest care according to institutional protocols.

Patients who remained unconscious following a sedation hold (sedation hold was defined as planned cessation or stepwise reduction of continuous sedative infusions to permit neurological examination once sedative effects were judged minimal and no ongoing neuromuscular blockade was present [13]) at ≥72 h post-arrest (defined as unconscious with a motor score ≤ 5 on the Glasgow Coma Scale) were enrolled into the formal neuroprognostication pathway [3,14]. Prior to publication of the 2025 guidance, eligibility for the pathway was based on the 2021 guideline threshold of a GCS motor score <3 at ≥72 h; this was subsequently updated to ≤5 to reflect the revised 2025 clinical assessment recommendation [15]. Only 11 patients were admitted after publication of the updated guidance and were therefore assessed under the revised ≤5 motor-score threshold. Patients who regained consciousness prior to this time point were managed outside the pathway and were not discussed at the neuroprognostication MDT.

### 2.3. Neuroprognostication Pathway

The neuroprognostication pathway was coordinated by a specialist neuroprognostication nurse and was based on principles outlined in contemporary European Resuscitation Council and European Society of Intensive Care Medicine guidelines for post-cardiac arrest care [3,9]. Key principles included delayed prognostication, multimodal assessment, and avoidance of reliance on isolated predictors of neurological outcome.

Neuroprognostication investigations were performed at the discretion of the treating clinical teams, guided by patient-specific clinical context, resolution of confounding factors (including sedation and metabolic derangement [13]), and investigation availability. Modalities included computed tomography (CT) of the brain, magnetic resonance imaging (MRI) of the brain, electroencephalography (EEG), somatosensory evoked potentials (SSEP), and serum neuron-specific enolase (NSE) measurement. Formal pupillometry was not available throughout the entire study period and was therefore not included in the present analysis.

In routine practice, CT brain was performed early when clinically indicated, including on admission or within the first 6–12 h in patients who failed to wake during sedation hold, developed seizures, or where the cause of cardiac arrest was uncertain. In this context, early CT was primarily diagnostic, to exclude alternative intracranial pathology or clarify the cause of arrest, rather than being used in isolation for prognostication. Repeat CT was commonly performed during the formal neuroprognostication window, particularly at 72–96 h, where assessment for evolving hypoxic-ischaemic brain injury was required. EEG was performed earlier where clinically indicated, including for suspected seizure activity, and was otherwise undertaken as part of formal multimodal prognostication. SSEP and NSE were generally undertaken once patients had entered the formal neuroprognostication window, after efforts had been made to minimise confounding from sedation, neuromuscular blockade, metabolic disturbance, or temperature-management effects. MRI brain was requested selectively, typically at 72–96 h, when CT findings were inconclusive and the patient remained unconscious, particularly where MRI findings were expected to contribute to MDT interpretation or subsequent management.

Table 1 summarises the guideline-recommended domains [15] of multimodal neuroprognostication and how these were implemented within the centre’s structured MDT pathway.

### 2.4. Multidisciplinary Neuroprognostication Meeting Structure

**Figure 1:**
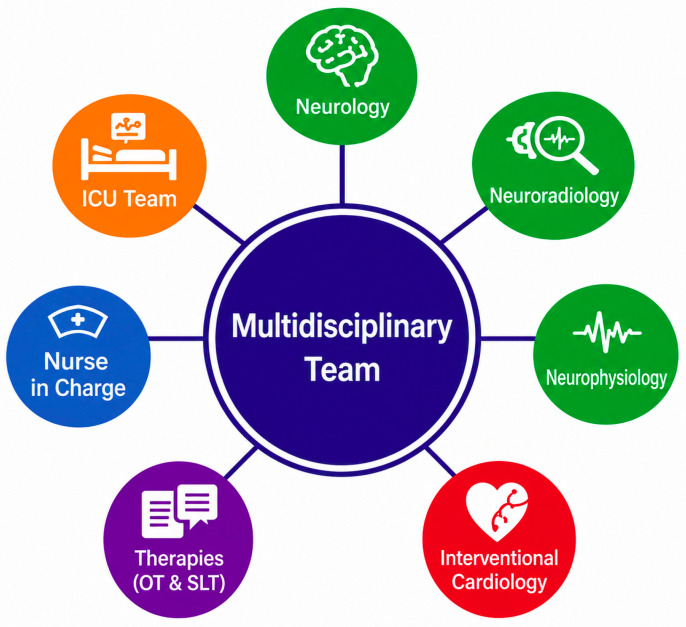
Multidisciplinary team structure for neuroprognostication after out-of-hospital cardiac arrest. The MDT included intensive care clinicians, neurology, neuroradiology, neurophysiology, interventional cardiology, specialist nursing, and therapy services, including occupational therapy (OT) and speech and language therapy (SLT).

Once relevant investigations were available, a formal MDT meeting was convened (see Figure 1 for a representation of specialist roles within the multidisciplinary team). MDT meetings were convened on an individual case basis once relevant clinical information and investigations were available, rather than according to a fixed schedule. This reflected variation in the number and timing of eligible OHCA patients. MDT review was recorded using structured documentation within the electronic patient record, which was uploaded to the clinical notes after the meeting.

**Figure 2:**
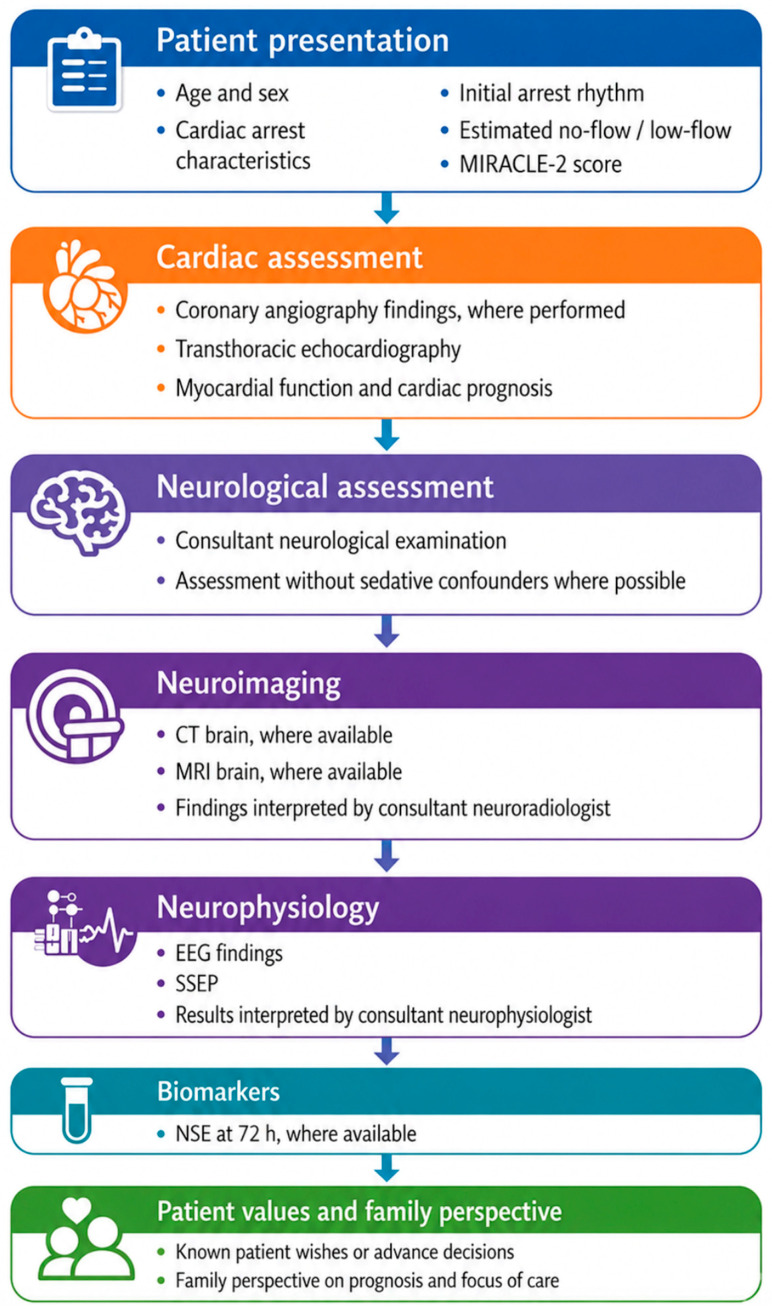
Domains reviewed during structured MDT neuroprognostication after cardiac arrest. The review incorporated patient and arrest characteristics, cardiac assessment, neurological examination, neuroimaging, neurophysiology, biomarkers, and patient values/family perspectives.

The MDT followed a structured format shown in Figure 2.

### 2.5. Decision-Making Process

Following presentation of all available clinical and investigative data, findings were interpreted collectively using the ERC neuroprognostication algorithm [3].

The MDT functioned as an independent expert advisory group, separate from routine clinical care team, although responsible treating clinicians were invited to attend to contribute relevant clinical context. Its role was to provide a consensus recommendation based on specialised interpretation of the available prognostic evidence, rather than to make a unilateral treatment decision.

Decisions were made only after consideration of the full multimodal assessment, with particular emphasis on concordance between multiple predictors of neurological outcome and exclusion of confounding factors where possible.

MDT recommendations were subsequently discussed with the patient’s family by the treating ICU team. Final decisions regarding ongoing treatment or WLST were made following shared decision-making discussions with family members, informed by MDT recommendations and the patient’s known wishes where available.

### 2.6. Data Collection

Clinical, anonymised data were extracted from our regional BCIS OHCA registry (REC (22/NW/0146)/CAG (22/CAG/0106)).

Collected variables included demographics, cardiac arrest features, MIRACLE-2 score [16,17], investigations performed (CT, MRI, EEG, SSEP, NSE), MDT outcomes, survival status and neurological outcome using the Cerebral Performance Category (CPC) scale [18].

Good neurological outcome was defined a priori as CPC 1–2, with poor neurological outcome defined as CPC 3–4 [18].

### 2.7. Outcome Measures

The primary outcome was survival to hospital discharge among adults admitted to ICU following OHCA. Neurological outcome at hospital discharge was assessed using the CPC score and dichotomised among hospital survivors as favourable neurological outcome (CPC 1–2) or unfavourable neurological outcome (CPC 3–4). Where analyses included death, CPC 5 was included within unfavourable outcome.

Secondary outcomes were the proportion of patients entering the formal neuroprognostication pathway, mode of death, utilisation of neuroprognostication investigations, number of prognostic modalities performed, and timing of WLST. Mode of death was categorised as death due to cardiorespiratory deterioration or WLST following MDT-supported assessment of poor neurological prognosis.

### 2.8. Statistical Analysis

Continuous variables are presented as mean (SD) or median (IQR), as appropriate, and categorical variables as counts and percentages. *p* values comparing patients who did and did not enter the neuroprognostication pathway were calculated using Mann–Whitney U tests for continuous variables and Fisher’s exact or chi-squared tests for categorical variables, as appropriate. No multivariable modelling was performed, given the descriptive study aim and clinician-mediated nature of treatment-limitation decisions. Analyses were performed using Excel (Microsoft), jamovi 2.7.16 and Python 3.14.6. Statistical significance was defined as a two-sided *p* value < 0.05. Missing data were not imputed. Missing data were limited to one missing MIRACLE2 score, one missing initial rhythm, and one missing CPC score at hospital discharge. Neuroprognostication investigation data were complete; investigations not performed were recorded as not performed rather than treated as missing.

## 3. Results

Between June 2022 and December 2025, a total of 406 patients were admitted to the CTC following OHCA. Of these, 310 were admitted to ICU for post-arrest care and comprised the study cohort; the remainder were admitted to the high dependency cardiology ward.

The cohort admitted to ICU (*n* = 310) was predominantly male (82.3%, *n* = 255), with females comprising 17.7% (*n* = 55). The mean age was 63.8 years (SD 13.7), with a median age of 64.0 years (IQR 54.2–75.0; range 17–90 years). The initial cardiac arrest rhythm was most commonly ventricular fibrillation (VF) in 82.9% (*n* = 257) of cases, followed by pulseless electrical activity (PEA) in 5.8% (*n* = 18), shockable rhythm detected by AED in 4.8% (*n* = 15), asystole in 4.2% (*n* = 13), and pulseless ventricular tachycardia (VT) in 1.9% (*n* = 6). Presenting rhythm was not available for one patient. Baseline characteristics of the overall ICU cohort, stratified by neuroprognostication pathway status, are shown in Table 2.

Overall, 61.6% (*n* = 191) patients admitted to ICU did not undergo neuroprognostication. Of these, 75.9% (*n* = 145) were conscious with a motor score ≥5 at 72 h and were discharged without requiring neuroprognostication, while the other 24.1% (*n* = 46) died due to cardiorespiratory deterioration before 72 h in ICU. Among the 145 patients discharged without requiring formal neuroprognostication, 98.6% (*n* = 143) had a favourable neurological outcome at hospital discharge (CPC 1–2), while 1.4% (*n* = 2) had an unfavourable outcome (CPC 3–4). The detailed CPC breakdown in this group was 85.5% (*n* = 124) with CPC 1, 13.1% (*n* = 19) with CPC 2, and 1.4% (*n* = 2) with CPC 3.

**Figure 3:**
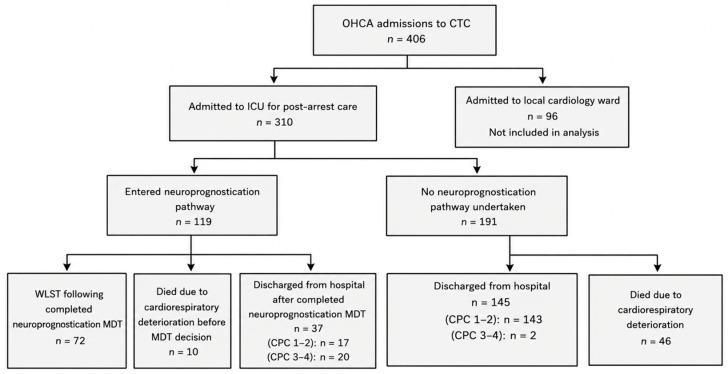
Patient flow through the ICU and neuroprognostication pathway following out-of-hospital cardiac arrest.

Overall, 38.3% (*n* = 119) of patients entered the neuroprognostication pathway due to being unconscious with a motor score ≤5 at 72 h or later. 10 of these patients died before MDT decision-making, 72 underwent WLST after MDT review, and 37 survived to hospital discharge after a decision to continue life-sustaining treatment. Among these discharged survivors, 45.9% (*n* = 17) had a favourable neurological outcome at hospital discharge (CPC 1–2), while 54.1% (*n* = 20) had an unfavourable outcome (CPC 3–4). The overall cohort is summarised in Figure 3.

### 3.1. Patients Discharged from Hospital

**Figure 4:**
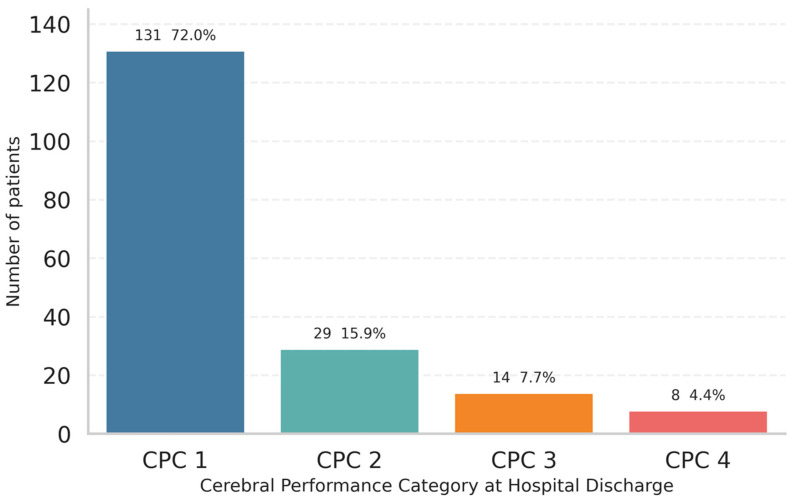
Neurological outcome at hospital discharge by Cerebral Performance Category. Bar chart showing the distribution of CPC scores at hospital discharge among patients surviving to hospital discharge. CPC 1–2 indicates favourable neurological outcome, while CPC 3–4 indicates unfavourable neurological outcome. Values are presented as *n* (%).

Among all patients admitted to ICU, 58.7% (*n* = 182) were discharged alive from hospital. Among discharged patients, 87.9% (*n* = 160) achieved a favourable neurological outcome (CPC 1–2), while 12.1% (*n* = 22) had an unfavourable outcome (CPC 3–4). The detailed CPC breakdown showed 72.0% (n = 131) with CPC 1, 15.9% (*n* = 29) with CPC 2, 7.7% (*n* = 14) with CPC 3, and 4.4% (*n* = 8) with CPC 4. This is shown in Figure 4.

Among discharged patients, the median age was 61.0 years (IQR 52.0–70.8; range 17–90 years). Age did not differ between the favourable and unfavourable outcome groups (Mann–Whitney U = 1772.5, *p* = 0.882).

Among patients discharged alive from hospital, 20.3% (*n* = 37) underwent formal neuroprognostication. CT head imaging was performed in 100.0% (*n* = 37), NSE testing in 89.2% (*n* = 33), EEG in 59.5% (*n* = 22), MRI in 51.4% (*n* = 19), and SSEP in 51.4% (*n* = 19). Within this cohort, 45.9% (*n* = 17) had a favourable neurological outcome at hospital discharge (CPC 1–2), while 54.1% (*n* = 20) had an unfavourable outcome (CPC 3–4).

Compared with patients who woke and were discharged without requiring MDT neuroprognostication, those undergoing formal neuroprognostication had higher admission MIRACLE2 scores [16,17] (median 4.0, IQR 3.0–4.0 vs. 2.0, IQR 1.0–4.0; *p* < 0.001), while age, sex, and presenting rhythm were broadly similar between groups.

### 3.2. Death on ICU Due to Cardiorespiratory Deterioration

Of the 310 patients included in the study, 18.1% (*n* = 56) died in ICU due to cardiorespiratory deterioration, before or during neuroprognostication. The mean age of deceased patients was 68.7 years (SD 14.3), with a median age of 72.0 years and a range of 17–89 years.

Patients who died due to cardiorespiratory deterioration in ICU were significantly older than those who survived to discharge (mean age 68.7 vs. 61.4 years; Mann–Whitney U = 6835.0, *p* < 0.001). Deceased patients also had significantly higher MIRACLE2 scores [16,17] compared with discharged patients (mean 5.44 vs. 2.75; Mann–Whitney U = 8690.0, *p* < 0.001), indicating worse predicted outcomes at presentation.

### 3.3. Patients Undergoing Withdrawal of Life-Sustaining Treatment

A total of 72 patients underwent WLST through the neuroprognostication MDT. Patients undergoing WLST had a median age of 68.5 years (IQR 59.8–76.0), and 73.6% (*n* = 53) were male. CT head imaging was performed in 100% (*n* = 72) of patients. NSE testing was performed in 91.7% (*n* = 66). Neurophysiological investigations were also widely utilised, with EEG performed in 90.3% (*n* = 65) and SSEP in 88.9% (*n* = 64) of patients. In contrast, MRI brain was used more selectively, when CT was inconclusive and was performed in 27.8% (*n* = 20) of patients.

**Figure 5:**
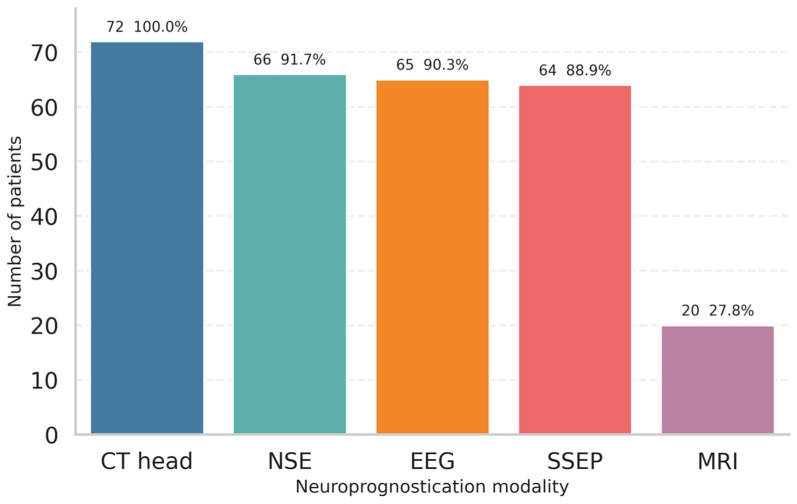
Individual neuroprognostication modalities in patients who underwent withdrawal of life-sustaining treatment. Bar chart showing the number and proportion of patients who underwent each neuroprognostication modality prior to WLST. Values are presented as *n* (%). CT, computed tomography; NSE, neuron-specific enolase; EEG, electroencephalography; SSEP, somatosensory evoked potentials; MRI, magnetic resonance imaging; WLST, withdrawal of life-sustaining treatment.

Overall, 95.8% (*n* = 69) of patients undergoing WLST received multiple neuroprognostication modalities (≥2). Four or more modalities were utilised in 87.5% (*n* = 63) of patients, while 20.8% (*n* = 15) underwent all five modalities assessed (CT, MRI, EEG, SSEP, and NSE). These results are shown in Figure 5.

The timing of WLST clustered within the first week following admission, with a median time to WLST of 5.5 days (IQR 4.0–8.0).

## 4. Discussion

In this single-centre cohort of adults admitted to a tertiary ICU following OHCA, we describe both overall patient outcomes and the operation of a structured MDT neuroprognostication pathway embedded in routine post-cardiac arrest care. Several findings are noteworthy. First, survival to hospital discharge was high in this selected CAC population, likely, due to adoption of the BCIS conveyance algorithm [12], with most survivors achieving favourable neurological outcomes. Second, among patients who underwent WLST for poor neurological prognosis, neuroprognostication was consistently based on delayed and multimodal assessment rather than isolated investigation findings. Third, decisions regarding WLST were typically made within the first week of ICU admission, after completion of a structured MDT review process.

The overall survival observed in this cohort is higher than that reported in many unselected OHCA populations [2,19,20]. This finding should be interpreted in the context of patient selection. Patients were admitted via the BCIS conveyance pathway to a tertiary cardiac arrest centre, resulting in a cohort enriched for shockable rhythms, suspected reversible cardiac causes, and access to specialist post-cardiac arrest care [11,12]. In this study, 82.9% of ICU-admitted patients had ventricular fibrillation as the initial rhythm. Therefore, the reported survival-to-discharge rate and favourable neurological outcome rate among survivors should not be interpreted as representative of general OHCA populations. Rather, these data provide contemporary benchmark information for comparable tertiary cardiac arrest centres managing similarly selected patients.

The central contribution of this study is the detailed description of how neuroprognostication was operationalised within a formal MDT framework. In patients who ultimately underwent WLST, uptake of neuroprognostication investigations was high, and nearly all patients underwent assessment using multiple modalities. CT brain imaging, EEG, SSEP, and NSE were widely utilised, while MRI was used more selectively [21]. This pattern likely reflects pragmatic considerations such as patient stability, access, and the incremental value of MRI in cases where uncertainty remained after other investigations [21]. Overall, these findings suggest that a structured MDT pathway can support consistent implementation of guideline-concordant multimodal prognostication in everyday ICU practice [3].

The timing of WLST is particularly important in post-cardiac arrest care because of the recognised risk of self-fulfilling prophecy [22]. In this cohort, WLST decisions clustered around day 5 after admission, which is consistent with a delayed approach allowing time for sedation washout, serial neurological assessment, and completion of multimodal investigations. However, in the absence of a comparator cohort or formal assessment of prognostic test performance, these data cannot determine whether the pathway reduced premature WLST decisions or improved prognostic accuracy.

An additional strength of the MDT pathway is that it formalises input from multiple specialties that each contribute distinct, but complementary expertise. Neurologists, neuroradiologists, neurophysiologists, intensivists, cardiologists, specialist nurses, and therapists all contribute to the interpretation of prognosis in a clinically and ethically complex setting. Such a model provides a structured framework through which multiple specialties can contribute to prognostic interpretation and communication with families; however, the present study was not designed to determine whether this improves consistency of practice, clinician decision-making, or family-centred outcomes [23]. Although family-centred outcomes were not measured in this study, a structured MDT process may also help families by grounding discussions in a transparent and sequential assessment pathway [23,24].

This study should be interpreted considering several limitations. It is a retrospective observational study from a single centre and therefore reflects local case mix, referral patterns, and service organisation. The cohort was drawn from a tertiary CAC and was heavily weighted toward shockable rhythms, limiting generalisability to non-specialist centres or unselected OHCA populations. The cohort was enriched for patients with shockable rhythms and suspected reversible cardiac causes; therefore, the observed survival and neurological outcomes should not be interpreted as representative of an unselected OHCA population. Neurological outcome was assessed at hospital discharge using CPC, which, although widely used, does not fully capture longer-term functional, cognitive, or quality-of-life outcomes [25]. In addition, while this study describes practice within a formal MDT pathway, it was not designed to assess the prognostic performance of individual modalities or to determine whether MDT involvement itself improves survival or neurological recovery [25,26]. The study was descriptive and did not include a comparator period or comparator centre; therefore, no causal inference can be made regarding the effect of the MDT pathway on survival, neurological outcome, timing of WLST, or consistency of decision-making. Accordingly, the findings should be interpreted as a descriptive account of pathway implementation and associated outcomes, rather than evidence that the MDT pathway improved clinical outcomes or decision-making processes. The eligibility threshold for formal neuroprognostication changed during the study period, from a GCS motor score <3 to ≤5 at ≥72 h, following publication of updated guidance [15]. However, only 11 patients were admitted after this update, precluding meaningful comparison of patient selection, investigation utilisation, or outcomes between eligibility definitions. Future evaluation within our service will be required to assess the operational impact of the revised threshold.

Nevertheless, these data provide an important real-world account of how structured neuroprognostication can be embedded into routine ICU care after OHCA. The study moves beyond simple reporting of survival and CPC outcomes by showing how use of investigations, multidisciplinary review, and WLST decision-making were integrated within a pathway designed to align with contemporary neuroprognostication guidance. This is likely to be of practical relevance to other centres seeking to develop or benchmark similar services.

Future work should focus on prospective evaluation of structured neuroprognostication pathways, including longer-term neurological, cognitive, and quality-of-life outcomes, as well as comparative studies across centres with different organisational models. Further research should also explore family experience and the impact of formal MDT processes on communication, consistency of decision-making, and perceived quality of care.

## 5. Conclusions

In this tertiary CAC cohort, overall survival to hospital discharge was high and neurological outcomes among survivors were predominantly favourable. Within this setting, delayed, multimodal neuroprognostication and WLST decision-making were delivered through a structured MDT pathway aligned with contemporary guideline recommendations. These findings provide contemporary real-world benchmark data for comparable tertiary cardiac arrest centres caring for similarly selected OHCA populations and seeking to evaluate or develop similar neuroprognostication pathways.

## Figures and Tables

**Table 1:** Guideline-recommended neuroprognostication domains and centre practice.

Guideline Recommended Domain	Centre Practice	Advantages	Limitations
Delayed assessment after exclusion of confounders	Patients remaining unconscious after sedation hold at ≥72 h entered the pathway.	Reduces risk of premature prognostication.	Residual confounding may persist in critically ill patients.
Multimodal assessment	MDT review incorporated clinical context, neurological assessment, imaging, neurophysiology, biomarkers, and family/patient values.	Avoids reliance on a single predictor.	Requires coordination across specialties.
CT brain	Available and routinely used; performed in all patients undergoing WLST.	Widely available and feasible in ICU patients.	May be less sensitive than MRI for some hypoxic-ischaemic injury.
MRI brain	Used selectively when further clarification was required.	Can provide additional information when uncertainty remains.	Limited by patient stability, logistics, and scanner access.
EEG	Used as part of neurophysiological assessment.	Assesses brain activity and seizure patterns.	Can be affected by sedation and requires expert interpretation.
SSEP	Used as part of neurophysiological assessment.	Complements EEG and imaging findings.	Requires specialist neurophysiology availability.
Serum NSE	Used as the biomarker component of assessment.	Adds a biochemical measure to multimodal assessment.	Interpretation depends on timing and clinical context.
Quantitative pupillometry	Not available throughout the study period and not included in the analysis.	May reduce subjectivity where available.	Not consistently implemented during the study period.

Abbreviations: CT, computed tomography; EEG, electroencephalography; ICU, intensive care unit; MDT, multidisciplinary team; MRI, magnetic resonance imaging; NSE, neuron-specific enolase; SSEP, somatosensory evoked potentials; WLST, withdrawal of life-sustaining treatment.

**Table 2:** Baseline characteristics and outcomes stratified by neuroprognostication pathway status.

Variable	Overall Cohort *n* = 310	No Neuroprognostication *n* = 191	Neuroprognostication Pathway *n* = 119
Age, years, median (IQR)	64.0 (54.2–75.0)	64.0 (54.5–74.5)	65.0 (54.5–75.0)
Age, years, mean (SD)	63.8 (13.7)	63.7 (13.7)	64.1 (13.7)
Male sex, *n* (%)	255 (82.3)	160 (83.8)	95 (79.8)
MIRACLE2 score, median (IQR)	4.0 (2.0–5.0)	2.0 (1.0–4.0)	4.0 (3.0–4.0)
MIRACLE2 score, mean (SD)	3.7 (2.0)	3.3 (2.2)	4.5 (1.4)
Shockable VT/VF	263 (84.8)	167 (87.4)	96 (80.7)
Non-shockable/other	47 (15.2)	24 (12.6)	23 (19.3)
Discharged alive, *n* (%)	182 (58.7)	145 (75.9)	37 (31.1)
Withdrawal of life-sustaining treatment, *n* (%)	72 (23.2)	0 (0.0)	72 (60.5)
Death due to cardiorespiratory deterioration, *n* (%)	56 (18.1)	46 (24.1)	10 (8.4)

Data are presented as median IQR, mean SD, or *n* %. Neuroprognostication pathway status was defined according to entry into the MDT neuroprognostication pathway. Shockable rhythm was defined as VT or VF; all other rhythms were classified as non-shockable/other.

## Data Availability

All data used in this study were obtained from the British Cardiovascular Intervention Society (BCIS) Out-of-Hospital Cardiac Arrest (OHCA) registry, coordinated from the Essex Cardiothoracic Centre. No new primary data were generated for the purposes of this study. Access to the BCIS OHCA registry is governed by national and institutional approvals, and the underlying individual-level data cannot be made publicly available. Aggregated data supporting the findings of this study are available from the corresponding author upon reasonable request and subject to appropriate approvals.

## Abbreviations

AED, automated external defibrillator; BCIS, British Cardiovascular Intervention Society; CAC, cardiac arrest centre; CPC, Cerebral Performance Category; CT, computed tomography; EEG, electroencephalography; ERC, European Resuscitation Council; ESICM, European Society of Intensive Care Medicine; GCS, Glasgow Coma Scale; ICU, intensive care unit; IQR, interquartile range; MDT, multidisciplinary team; MRI, magnetic resonance imaging; NSE, neuron-specific enolase; OHCA, out-of-hospital cardiac arrest; ROSC, return of spontaneous circulation; SD, standard deviation; SSEP, somatosensory evoked potentials; STEMI, ST-elevation myocardial infarction; WLST, withdrawal of life-sustaining treatment.
